# Understanding the gut microbiome through a fitness intervention of aerobic and resistance training for individuals with type 2 diabetes mellitus (GUTFIT: A Study Protocol)

**DOI:** 10.1371/journal.pone.0343294

**Published:** 2026-02-23

**Authors:** Amy M. Thomson, Dominique C. Drost, Neil M. Johannsen, Cristoforo Silvestri, Martin Sénéchal

**Affiliations:** 1 Cardiometabolic Exercise & Lifestyle Laboratory, Fredericton, New Brunswick, Canada; 2 Faculty of Kinesiology, University of New Brunswick, Fredericton, New Brunswick, Canada; 3 School of Kinesiology, Louisiana State University, Baton Rouge, Louisiana, United States of America; 4 Pennington Biomedical Research Center, Baton Rouge, Louisiana, United States of America; 5 Faculté de Médecine, Université Laval, Laval, Québec, Canada; 6 Institut Universitaire de Cardiologie et de Pneumologie de Québec, Québec City, Québec, Canada; PLOS: Public Library of Science, UNITED KINGDOM OF GREAT BRITAIN AND NORTHERN IRELAND

## Abstract

**Introduction:**

Exercise is a cornerstone of type 2 diabetes (T2DM) management, yet individuals exhibit vast inter-individual variability in glycemic response to interventions. Gut microbial diversity and exercise intensity may be factors influencing this response variability. However, the interplay between exercise intensity, microbial adaptations, and glycemic outcomes in individuals living with T2DM remains unclear.

**Objectives:**

The purpose of this protocol is to describe the GUTFIT study, which aims to test whether performing vigorous-intensity combined aerobic and resistance training produces greater changes in glycemia and gut microbial diversity than moderate-intensity training in individuals living with T2DM. A secondary objective is to explore whether decreases in glycemia after exercise are associated with alterations in gut microbial community architecture and diversity.

**Methods:**

The GUTFIT Study (NCT06268743) is a parallel-group, single-blinded, randomized trial involving 40 adult participants (n = 20 female) living with T2DM. Participants will be randomized to 16 weeks of: 1) vigorous-intensity exercise (aerobic training at 70–80% heart rate reserve and resistance training at 8–10 repetitions of 75–80% maximal strength) or 2) moderate-intensity exercise (aerobic training at 45–55% heart rate reserve and resistance training at 12–15 repetitions of 65–70% maximal strength). Glycemia will be measured via glycated hemoglobin (HbA1c), and gut microbial composition will be determined in participant fecal samples using next-generation sequencing (Illumina MiSeq) of 16S ribosomal DNA genes. All outcome measures will be tested pre- and post-intervention.

**Discussion:**

Results of this study will provide further insight into the role of exercise intensity in changes in glycemia and the gut microbiome, and whether there is an intensity-dependent association between exercise-induced changes in glycemia and gut microbial diversity in individuals living with T2DM.

## Introduction

As global rates of type 2 diabetes mellitus (T2DM) and its related complications continue to rise, there is a growing need for more effective lifestyle interventions [1]. Exercise is a well-established intervention for managing T2DM, primarily due to its capacity to enhance insulin sensitivity, thereby improving glycemia and mitigating disease-related complications [2–4]. However, considerable inter-individual variability exists in the metabolic response to exercise. While some individuals demonstrate marked improvements in glycemia and insulin sensitivity, others show little to no benefit, despite comparable adherence to prescribed programs [5,6]. This variability underscores the need to identify modifiable factors that influence individual responsiveness to exercise interventions in an effort to optimize exercise prescriptions for T2DM management.

Several factors have been proposed to account for this response heterogeneity, including variation in training modality, intensity, volume, or duration, baseline metabolic health, and genetic predisposition [7]. Among these, exercise intensity has received particular attention, as it can be manipulated to potentially enhance exercise effectiveness [7,8]. Nevertheless, recent findings suggest that modifying aerobic exercise intensity alone does not consistently resolve non-responsiveness in glycemic outcomes among individuals with prediabetes or T2DM [6]. These observations suggest that other mechanisms may mediate the metabolic benefits of exercise.

The gut microbiome has gained recent attention as a key regulator of metabolism and glucose homeostasis, with potential implications for modulating individual responses to exercise [5,7,9–14]. Regular physical activity is associated with increased microbial diversity and a shift towards a microbiota profile enriched with beneficial taxa [15–20]. In individuals living with T2DM, these exercise-induced microbial alterations may have clinically relevant implications as they are linked to improved glycemia and reduced systemic inflammation, addressing key pathophysiological features of the disease [12,13,21,22].

Emerging evidence suggests a bidirectional relationship between exercise and the gut microbiome: while exercise alters microbial composition, baseline gut microbiota profiles may, in turn, modulate the glycemic response to exercise [5,13,23]. For instance, Liu et al. (2020) found that differential alterations in gut microbiota composition were strongly correlated with glycemic response to exercise in men living with prediabetes following an exercise intervention [5]. Their analysis further revealed that microbial capacity for metabolite production increased in exercise responders, and that distinct baseline gut microbial signatures predicted exercise responsiveness for glycemic outcomes [5]. These findings suggest that gut microbial profiles may serve as biomarkers of exercise responsiveness and offer novel insight into the mechanisms underlying inter-individual variability in metabolic outcomes.

Exercise modality and intensity have been proposed as factors that modulate exercise response in T2DM via gut microbiome alterations. Higher-intensity exercise has been associated with greater shifts in microbial diversity [24,25], which may amplify the production of short-chain fatty acids (SCFAs) and other metabolites that improve insulin sensitivity and glucose regulation [12,14]. In 2023, Torquati et al. showed that eight weeks of moderate-intensity continuous training and high-intensity interval training resulted in distinct changes in gut microbial community composition and the relative abundance of specific health-promoting taxa between the intensity groups [26]. However, these microbial adaptations did not translate into significant between-group differences in glycemic outcomes or gut-derived metabolites such as SCFAs, suggesting that the link between exercise intensity, gut microbial adaptations, and glycemic regulation remains equivocal [26]. Exercise modality can also induce distinct microbial modifications in the gut [9]. While aerobic exercise is well-documented for its ability to enhance the abundance of SCFA-producing bacteria, which are known to improve insulin sensitivity and reduce inflammation [16,23–25,27,28], resistance training may promote microbial diversity and complement aerobic exercise effects [23,24,29], suggesting that combined interventions could maximize the microbiota-mediated benefits for metabolic health [9,15,24,30,31].

To date, the majority of research surrounding exercise and the gut microbiome has focused on healthy populations, with limited data exploring the interplay between exercise intensity, microbial adaptations, and glycemic outcomes in individuals living with T2DM. Investigating these relationships could provide valuable insights into optimizing exercise prescriptions and addressing inter-individual variability in T2DM management. As such, the primary objective of this study is to determine whether performing combined aerobic and resistance training at a vigorous intensity improves glycemia, measured by hemoglobin A1c (HbA1c), and increases gut microbial diversity compared to performing combined training at a moderate intensity. We hypothesize that individuals exercising at a higher intensity will experience greater reductions in HbA1c and more significant alterations in gut microbial community structure, with greater increases in gut microbial diversity, than individuals training at a lower intensity.

## Materials and methods

### Study design

The GUTFIT Study is a parallel-group, single-blinded, randomized trial.

### Study setting

The GUTFIT study (NCT06268743) will be conducted in the Cardiometabolic Exercise and Lifestyle Laboratory (CELLAB) in the Faculty of Kinesiology at the University of New Brunswick in Fredericton, Canada. This location was selected for its private gym facility, availability of necessary equipment, and access to research staff who are experts in clinical trial delivery and trained to work with individuals living with T2DM.

### Eligibility criteria

#### Inclusion criteria.

1. Community-dwelling adults aged 19 years and older.
2. Currently living with T2DM, confirmed by a glycated hemoglobin (HbA1c) value of ≥ 6.5% or diagnosed by a physician with T2DM with an HbA1c of ≥ 5.7%.
3. Not currently partaking in regular physical activity, defined as 150 minutes of moderate-to-vigorous aerobic activity and two or more days of resistance training per week as per the 24-Hour Movement Guidelines [32], or averaging >10,000 steps per day over a five-to-seven-day period at baseline.

#### Exclusion criteria.

1. Diagnosed with low iron concentration, anemia, or currently being treated for these conditions.
2. Diagnosed with any red blood cell-altering conditions.
3. Diagnosed with any cardiovascular disease that would impact the ability to safely participate in exercise training.
4. Currently prescribed any medication that would impact the ability to use a heart rate monitor to accurately track exercise.
5. Diagnosed with any gut microbiome-altering conditions (e.g., Celiac disease, GERD, irritable bowel syndrome (IBS), or inflammatory bowel disease (IBD)).
6. Currently taking any gut microbiome-altering supplements (prebiotics/probiotics) or report taking antibiotics throughout the trial.
7. Unstable T2DM medication over the past 3 months.

### Recruitment

The GUTFIT study is currently recruiting participants from the city of Fredericton and the Greater Fredericton Area via social media and radio advertisements, as well as advertisements placed in pharmacies, healthcare centers, physician offices, and community organizations. Further recruitment is occurring through electronic communication, including e-newsletters within various organizations and groups. Participants from previous studies who expressed interest in being considered for future research have also been contacted. Recruitment began on February 29th, 2024, with the first participant enrolled on April 3^rd^, 2024. Recruitment is expected to be completed by March 2026, and the anticipated completion date of all data collection and follow-up visits is June 2026. Results from this study are anticipated to be available by January 2027.

### Intervention

Eligible participants for the GUTFIT study will be randomized into one of two intervention arms: 1) moderate-intensity exercise, or 2) vigorous-intensity exercise.

Participants in both intervention groups will be randomized and scheduled to begin exercise sessions within 1 week of completing all baseline testing. The 16-week training protocol requires participants to perform two resistance-training sessions per week and at least two aerobic exercise sessions per week. All exercise sessions will be supervised by research staff and take place in an exercise facility located in the CELLAB. To maximize adherence to the intervention, exercise sessions are scheduled on a weekly basis, with research staff available 7 days a week.

#### Resistance training.

The resistance training component of the intervention involves eight resistance exercises: seated chest press, triceps extension, lat pull-down, leg press, knee extension, and knee flexion using Atlantis Strength weight machines; seated shoulder press with dumbbells; and unweighted abdominal crunches. Resistance training sessions will occur twice per week on non-consecutive days. The moderate-intensity group will perform resistance training at 65–70% of the one-repetition maximum (1RM) for one set of 12–15 repetitions per exercise. The vigorous-intensity group will perform the resistance training at 75–80% of the 1RM for one set of 8–10 repetitions. Participants’ prescribed weight for an exercise will be increased if they successfully complete the maximum number of repetitions of an exercise in two consecutive sessions. Abdominal crunches will remain unweighted for the duration of the intervention. If a participant is unable to perform an abdominal crunch, they will start with seated chair crunches and progress to full abdominal crunches as they are able.

#### Aerobic exercise

For the aerobic exercise component of the intervention, participants will expend 10 kilocalories (kcal) per kilogram of body weight per week (KKW) using a treadmill (StarTrac) or a stationary bike (Ergoline). Participants will be eased into the program using a 2-week progressive start; they will burn 8KKW in Week 1 and 9KKW in Week 2. For the remaining 14 weeks (Weeks 3–16), participants will expend 10KKW per week. Participants will be allowed to choose the number of sessions to complete their required aerobic exercise, with a minimum of two days of exercise required and whether they complete a week of exercise using the treadmill or the bike. The speed and grade of the treadmill or the wattage and revolutions per minute (RPM) of the bike throughout the session will be determined by the participant as long as the prescribed intensity is achieved and maintained for the duration of each session. If necessary, the supervising research staff will instruct participants to adjust the speed or grade or watts or RPM to maintain the prescribed intensity. The moderate-intensity group will perform aerobic exercise at 45–55% of heart rate reserve (HRR), and the vigorous-intensity group will perform aerobic exercise at 70–80% of HRR. Participants will be given 5 minutes to warm up and reach target intensity at the beginning of each session, and 5 minutes for an active cool-down at the end of each session, neither of which will be counted toward their weekly caloric expenditure. To account for changes in cardiorespiratory fitness and ensure participants train at the appropriate intensity for their respective group, a new resting heart rate value will be measured at the start of every fifth week (Week 5, 9, 13) to account for changes in HRR.

#### Caloric expenditure.

Caloric expenditure will be calculated using the American College of Sports Medicine (ACSM) treadmill and stationary bike equations. Participants’ weight will be recorded in kilograms at the beginning of each week and entered into training software to determine the total kcals that need to be expended for that week. Throughout each aerobic session, the participant’s speed and grade for treadmill, or wattage and RPM for stationary bike, will be recorded every 5 minutes to calculate and track caloric expenditure and to determine the time required per session.

#### Exercise monitoring.

To ensure participants are exercising at the appropriate intensity, research staff will supervise each session and record participant heart rate every 5 minutes using the Polar Team2 (Polar, Kempele, Finland) system, along with treadmill speed and slope or bike wattage and RPM. The supervising staff will ensure that participants’ heart rates remain within the prescribed intensity range, adjusting treadmill or bike settings as necessary.

#### Resting heart rate re-evaluation.

**Fig 1:**
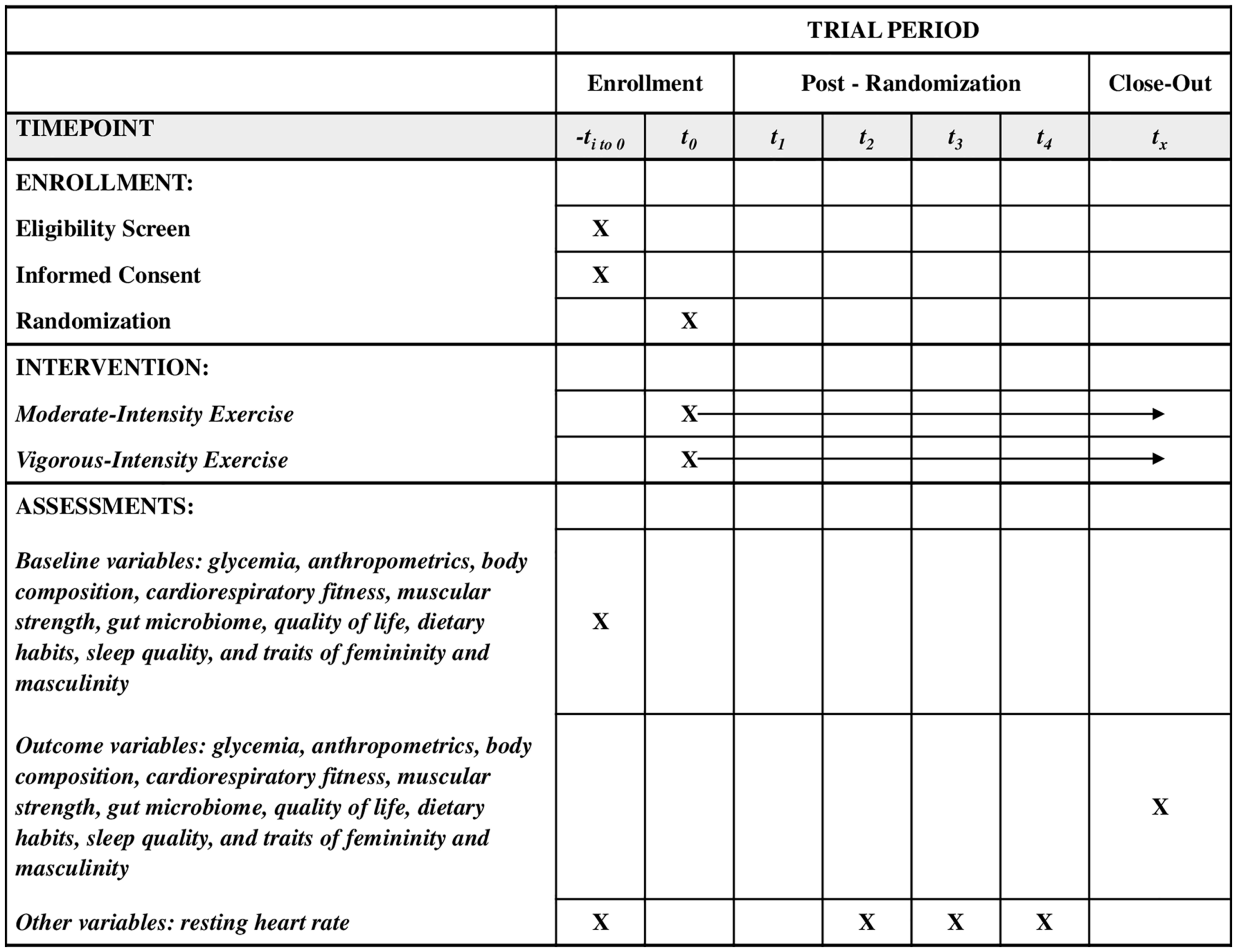
SPIRIT Participant timeline: Schedule of enrollment, interventions, and assessments. t_0_: time of randomization, t_1_: weeks 1-4 of intervention, t_2_: weeks 5-8 of intervention, t_3_: weeks 9-12 of intervention, t_4_: weeks 13-16 of intervention.

Resting heart rate (RHR) will be recorded at baseline testing and re-evaluated after every fourth week throughout the intervention (assessed at the beginning of the first session of every fifth week: Week 5, Week 9, Week 13) (Fig 1). As RHR will be used to prescribe intensity-dependent target heart rate ranges for the aerobic portion of the intervention, multiple measurements will be taken to find the average. Participants will be fitted with a Polar FT1 heart rate monitor (Polar, Kempele, Finland) worn around the chest and asked to lie supine on a massage table for a period of 10 minutes. Following five minutes of rest, heart rate, in beats per minute, will be recorded every minute for the next five minutes. These values will then be averaged and recorded as the new RHR. If the heart rates recorded differ by greater than five beats per minute, an additional five minutes of heart rate data will be recorded and used for this average.

### Deviations from protocol

Research staff will emphasize that each participant completes their required 10KKW and both sessions of resistance training each week. If a participant is absent from the trial for a full week (e.g., due to illness or a family emergency), an additional week will be added at the end of the trial for each week missed. The reason provided for missing a week of training will be documented and available for interpretation and analysis purposes. If a participant is unable to complete the required KKW of aerobic training after starting a week of exercise (illness, injury, emergency, etc.), this will be handled as follows: 1) if the participant completes less than one full session or less than one third of their caloric expenditure for that week, the completed session will be omitted and that week restarted when the participant returns, or 2) if the participant has completed more than 50% of their required caloric expenditure for the week but cannot complete the final session, the remaining calories will be carried over into the following week of the trial. Enrollment in the trial will be discontinued if a participant experiences an injury or medical event that would limit safe participation or require medical attention, or if the participant receives medical advice to withdraw from the trial.

### Data collection and management

At the time of initial contact with research staff, participants will be assigned a unique identifier (ID), and all files will be deidentified. Participants will meet with research staff for the purpose of data collection a total of four times: twice at baseline testing and twice at post-testing, in addition to the weekly exercise sessions. All data obtained from baseline and post-intervention testing visits will be collected in written form and subsequently entered into electronic files. All other data collected throughout the intervention will be collected electronically. Physical copies of files will be stored locally in a locked cabinet within a locked room in a restricted research lab at the University of New Brunswick, while digital copies will be password-protected and stored on a secure server operated by the University of New Brunswick.

The level of risk for adverse events associated with participation in GUTFIT is considered low. If an adverse event occurs, it will be recorded and reported to the principal investigator, who will inform the Research Ethics Board.

### Outcomes and instrumentation

#### Primary outcome.

The primary outcome of the GUTFIT study is glycated hemoglobin (HbA1c). A finger prick will be conducted using a Safe-T Pro Plus single-use lancet (Accu-Chek, Roche Diagnostics, Switzerland) to collect a 1 μL sample of whole blood. The sample will then be analyzed using a DCA Vantage Analyzer (V 4.4.0.0, Siemens Healthineers, Oakville, Ontario, Canada), a valid and accurate measure of HbA1c with intra-assay coefficients of variance of 1.55-2.29% [33], where rapid assessment will be conducted. For the purpose of these analyses, a significant change in HbA1c will be considered a decrease equal to or exceeding the minimal clinically important difference (MCID) of 0.3% [34,35] following participation in the trial.

#### Secondary outcomes.

Demographics, family medical history, and current medication usage will be recorded by research staff for all participants during baseline testing. Participants will be monitored throughout the study and asked to report any changes in medication use to research staff. Medication usage will be confirmed through detailed label inspection or a pharmacy printout at baseline and post-testing. To confirm and record participants’ physical activity levels for eligibility criteria, participants will complete the Get Active Questionnaire [36] and wear a Fitbit Charge 3 pedometer (Fitbit Inc., San Francisco, California, USA) for a 5–7-day period between the first and second baseline testing visits, over which their daily step count will be averaged, and the participant eligible if the average is < 10,000 steps per day.

Anthropometric and physiological measurements will occur at each time point over the span of two days, separated by approximately one week. Participants’ height, weight, body mass index (BMI), blood pressure, resting heart rate, and hip and waist circumference will be measured by a member of the research staff in accordance with the Canadian Society for Exercise Physiology protocols [37]. Briefly, participants’ height and weight will be measured to the nearest 0.5 cm and 0.1 kg, respectively, using a stadiometer and calibrated column scale (SECA707, SECA, Mt. Pleasant, USA). For the height measurement, participants will be asked to stand straight with their feet together, arms at their sides, with no shoes, and the measurement will be taken following an inhalation [37]. BMI will be calculated from the height and weight measurements (BMI = kg/m^2^). Waist circumference will be measured at the upper lateral border of the iliac crest at the end of a normal expiration while the participant stands with their feet shoulder-width apart. The average of two measurements, to the nearest 0.5 cm, will be recorded with an anthropometric tape measure [37]. If the two measures differ by greater than 1 cm, a third measure will be performed, and the average of the two closest measures will be recorded. Hip circumference will be measured around the maximal circumference of the buttocks, following the same protocol as waist circumference [38]. Resting heart rate and blood pressure will be measured twice while the participant is seated, following at least five minutes of rest, using an Omron digital blood pressure monitor (OMRON Healthcare Co., Ltd., Kunotsubo, Terado-cho, Muko, Kyoto, 617−0002, Japan) [37]. The average of the two measures will be recorded; if the average is ≥ 150/90 mmHg, the participant will remain seated for an additional five minutes of rest, and the measures will be repeated.

Body composition, including fat mass, lean mass, and body fat percentage, will be estimated using dual-energy x-ray absorptiometry (DXA) using a Hologic Horizon® DXA System (Hologic Canada ULC, Mississauga, ON, Canada). Participants will present to the CELLAB following a 12-hour overnight fast and will be asked to refrain from exercising for 24 hours prior to testing. Participants will also be instructed to wear loose-fitting clothing with no metal (buckles, zippers, etc.), lie supine on the DXA table, and remain still for the duration of the scan. Arms will be placed at the participants’ sides, with palms facing medially and thumbs pointing upward. The coefficient of variation in the CELLAB is 0.6% for lean mass and 0.7% for body fat percentage. This was performed on 33 individuals (males, n = 10) with a mean age of 23.4 years and a mean body mass index (BMI) of 25.6 kg/m^2^.

Cardiorespiratory fitness will be assessed using a modified Balke and Ware treadmill test protocol with a progressive start. Participants will walk at a speed of 4.5 km/h at a 0% grade on a treadmill (9500HR (Life Fitness, Illinois, USA)) as a warm-up. After 2 minutes, the speed will be increased to 5 km/hr, and the grade will increase to 2.5% (minutes 2–4). Following another 2 minutes, participants’ speed will be increased to 5.5 km/h and grade to 5.0% (minutes 4–6). Grade will then progressively increase by 1.0% every minute until a grade of 15% is achieved. If the participant has not reached maximal capacity, the speed will increase by 0.8 km/h each minute until test termination criteria are met. Following the completion of the test, participants will be instructed to continue walking at a decreased speed and grade for 5 minutes of active recovery. The participant will then have 3 minutes of seated passive recovery. Gas exchange will be continuously monitored during the test using a TrueOne 2400 Metabolic Cart (ParvoMedics, Salt Lake City, Utah, USA), heart rate data will be obtained over the course of the test using the Polar FT1 heart rate monitor (Polar, Kempele, Finland), and blood pressure data will be recorded every 2 minutes using a SunTech® Tango M2 Automated Blood Pressure Monitor (SunTech Medical, Inc., Morrisville, North Carolina, USA). VO_2peak_ data will be calculated as an average of the six highest five-second measures (30 seconds) during the last minute of the test.

Muscular strength will be assessed through 1-repetition maximum (1-RM) testing for each of the weighted exercises performed throughout the program, excluding abdominal crunches. Participants will perform two warm-up sets: the first with a weight they can complete 6–10 repetitions with, and the second with a weight they can complete 3–5 repetitions with. Following the warm-up sets, research staff will increase the weight until participants can no longer lift the weight or show improper form during the movement. Rest periods between maximal attempts will be 1–3 minutes. There will be a maximum of seven attempts to reach the 1-RM. If the 1-RM is not reached, the participant will repeat testing for that exercise during their first exercise session, following the same protocol.

The gut microbiome will be analyzed using next-generation sequencing (Illumina MiSeq) of 16S ribosomal DNA genes. Participant fecal samples will be collected using OMNIgene®•GUT fecal sample collection kits (DNA Genotek Inc., Ontario, Canada) to ensure high-quality preservation of the microbial community structure. Participants will receive detailed instructions for collecting the sample and will be provided with a collection kit to use at home on the day before their first exercise session and after their last exercise session. Once collected, samples will be pipetted into microcentrifuge tubes and stored at −80 °C until the end of the intervention for batch analysis.

DNA from fecal samples will be extracted using the Qiagen DNeasy PowerSoil kit. The DNA concentrations of the extracts will be measured fluorometrically with the Quant-iT PicoGreen dsDNA kit on a Qubit (Thermo Fisher Scientific, Waltham, MA, USA), and the DNA samples will be stored at −20 °C until 16S rDNA library preparation is completed according to the Illumina “Preparing 16S Ribosomal RNA Gene Amplicons for the Illumina MiSeq System” protocol. Briefly, 15 ng of DNA will be used as the template, and the V3–V4 region of the 16S rRNA gene will be amplified by PCR using the following primers: 16S Amplicon PCR forward primer = 5′-TCGTCGGCAGCGTCAGATGTGTATAAGAGACAGCCTACGGGNGGCWGCAG- 3′ and reverse primer 5′-GTCTCGTGGGCTCGGAGATGTGTATAAGAGACAGGACTACHVGGGTATCTAATCC- 3′. Followed by a second PCR reaction to introduce indices (Sets A, B, C, D; Illumina, San Diego, CA, USA). The 16S metagenomic libraries will be eluted in 30 μL of nuclease-free water, and 1 μL will be qualified with a Bioanalyser DNA 1000 chip (Agilent Technologies, Santa Clara, CA, USA) to verify the amplicon size (expected size ∼600 bp) and then quantified with a Qubit (Thermo Fisher Scientific, Waltham, MA, USA). The libraries will then be normalized and pooled to 2 nM, denatured, diluted to a final concentration of 10 pM, and supplemented with 5% PhiX control (Illumina). Sequencing (2 × 300 bp paired-end) will be performed using the MiSeq reagent kit V3 (600 cycles) on an Illumina MiSeq system (Illumina, San Diego, CA, USA). Image analysis and base calling will be carried out directly on the MiSeq. The preprocessing of obtained sequences and bacterial taxa assignation will be performed according to the Dada2 pipeline (version 1.10.1) according to the recommended workflow [39] and ASV sequences assigned to taxonomy using the most recent SILVA taxonomic database (SILVA SSURef 138.1 NR, March 2021) as a reference dataset using default parameters [40,41]. Resulting sequence data will be processed using the MicrobiomeAnalyst platform [42]. To deal with potential large differences in sampling depth, rarefaction will be performed; otherwise, differences in sequencing depth will be normalized by cumulative sum scaling. Microbiota composition will be assessed by calculating beta-diversity indexes obtained using the Bray–Curtis index, and Principal Coordinate Analysis (PCoA) will be performed and analyzed using PERMANOVA analysis.

Short-chain fatty acids (SCFA) will be extracted and measured by gas chromatography. 100 μL of the fecal sample will be mixed with 1 mL of ultrapure water and homogenized using a Bead Ruptor (Omni, Kennesaw, Georgia) at 4.0 m/s for 2 minutes before centrifugation at 5500 × *g* for 30 minutes at 4 °C. From the resulting supernatant, 500 μL will be collected and processed according to a previously described method [43]. The SCFAs to be analyzed included acetic, propionic, butyric, isobutyric, valeric, and isovaleric acid. Quantification will be performed using gas chromatography with flame ionization detection (GC-FID; Shimadzu, Kyoto, Japan). Each sample will be analyzed in duplicate, and the average values calculated as relative proportions.

Quality of life, dietary habits, sleep quality, and traits of femininity and masculinity will be measured using questionnaires. Quality of life will be assessed using the 36-Item Short-Form Health Survey (SF-36), comprised of 8 domains scored from 0-100, with higher scores indicating better quality of life [44]. Diabetes-related quality of life will be assessed using the Audit of Diabetes-Dependent Quality of Life (ADDQOL), encompassing 19 domains scored from −9–3 with higher scores indicating a positive impact of diabetes [45]. Dietary habits will be recorded for a weekday and a weekend day using the ASA 24 [46], and eating behaviour will be assessed through the Three-Factor Eating Questionnaire-R-18 (TFER-R18) [47] with 18 items scored from 1-4 and summed into scale scores with higher scores indicating greater cognitive restraint, uncontrolled eating, and emotional eating. Sleep quality will be measured through the Pittsburgh Sleep Quality Index (PSQI) with 19 items summed into a global sleep score; a score of 5 or greater is indicative of poor sleep quality [48]. Traits of sex and gender will be recorded using BEM’s Androgyny [49] test using 60 traits rated on a 7-point Likert scale and the 5-Item Sex and Gender-Based Analysis Tool (SGBA-5) using self-perceived relations to 4 gendered aspects of health on a feminine to masculine continuum [50].

### Blinding

To maintain single blinding, participants will remain unaware of their randomization to the moderate- or vigorous-intensity exercise group for the duration of the intervention.

### Randomization

Randomization of the intervention participants will occur following completion of the second baseline testing visit using a 1:1 allocation ratio, stratified by sex, in a balanced randomized block design performed in sealed envelope software [51]. A member of the CELLAB staff who is not related to the project and has no contact with participants will hold the password-protected randomizations. When a new participant completes baseline testing and is ready for their first exercise session, the participant’s ID number and sex will be sent to the member of staff responsible for randomization via email, who will respond to research staff with the participant’s randomization (moderate or vigorous).

### Statistical analysis

The sample size calculation was based on an effect size of 0.30, an alpha of 0.05, and a power of 0.80. A sample size of n = 24 (n = 12 per group) was found to be appropriate to detect a significant difference between intensity groups. To account for a dropout rate of up to 40%, a total of 40 participants (20 per group) will be recruited. The primary outcome analyses will use the intention-to-treat principle, including all participants as randomized, and use a generalized linear mixed-effects model for repeated measures will be used. A *p* ≤ 0.05 will be considered significant.

### Ethics and dissemination

All experimental procedures have been approved by the Research Ethics Board at the University of New Brunswick (REB:2023–114). Any substantial protocol amendments will be submitted to the Research Ethics Board for review and approval prior to implementation.

### Informed consent

Upon initial contact with research staff, interested individuals will be provided with key information pertaining to the GUTFIT study and have their eligibility confirmed through a phone screening. Prior to the first baseline testing visit, eligible participants will be provided with a digital copy of the consent form to review. At the beginning of the first testing visit, eligible participants will have time to review a physical copy of the consent form, ask any questions, and consider their participation. If the participant decides to proceed with participating in the study, they will be asked to provide written consent, which will be cosigned by research staff. All participants are free to withdraw from the study at any time.

### Dissemination

Results from the GUTFIT study will be submitted to peer-reviewed journals and presented at scientific meetings. The findings from this study will be used to support and drive future randomized trials exploring the impact of exercise intensity on the gut microbiome in individuals living with T2DM. Participants will have the opportunity at the time of consent to request a copy of the study findings. Upon completion of the trial, a summary of findings and a personalized report will be provided to those participants.

## Supporting information

S1 File Clinical trial protocol. (PDF)

S2 File SPIRIT 2025 checklist. (PDF)
