# Supplementary material for: Understanding the gut microbiome through a fitness intervention of aerobic and resistance training for individuals with type 2 diabetes mellitus (GUTFIT: A Study Protocol)
Source: PLoS One. 2026 Feb 23;21(2):e0343294. doi: 10.1371/journal.pone.0343294 (PMC12928436; doi:10.1371/journal.pone.0343294)
Supplement: S1 File — (PDF) [file pone.0343294.s001.pdf]

Research Protocol

Background: Exercise is a well-established and effective intervention for managing chronic conditions like type 2 diabetes mellitus (T2DM), primarily due to its capacity to improve glycemia, enhance insulin sensitivity, and mitigate disease-related complications[1–3]. While exercise is widely beneficial, individuals response to exercise interventions vary greatly regarding glycemic outcomes[4,5]. As the global population grapples with rising rates of T2DM and its associated complications, identifying factors that influence individual responsiveness to exercise interventions is imperative for optimizing exercise prescriptions and improving health outcomes in individuals living with T2DM.

The gut microbiome has gained recent attention as a key regulator of metabolism and glucose homeostasis that may have a role in exercise response[4,6,7]. Evidence suggests that dysbiosis, an imbalance in gut microbiota composition and function, is both a contributor to and characteristic of T2DM through mechanisms such as impaired short-chain fatty acid (SCFA) production, increased gut permeability, and systemic inflammation[6,8,9]. While most research has focused on healthy or athletic populations, exercise has been shown to positively influence microbial diversity and function, potentially mediating the metabolic health benefits of exercise in chronic populations[6,10]. Emerging evidence suggests that exercise not only alleviates metabolic dysfunctions but also positively modulates the gut microbiome, enhancing diversity, and promoting the growth of beneficial taxa. These microbiome alterations may, in turn, amplify the metabolic benefits of exercise and create a bidirectional relationship [11]. For instance, Liu et al. (2020) found that changes in gut microbiota composition were strongly correlated with glycemic responses to exercise in men living with prediabetes[4]. Their sub-analysis further revealed that microbial diversity significantly increased in the exercise responders and that baseline microbial signatures were predictive of glycemic outcomes[4]. These findings suggest that gut microbial diversity may modulate glycemic response to exercise and potentially differentiate exercise responders from non-responders.

Exercise intensity and modality have also been proposed as factors to modulate exercise response in T2DM, with potential implications for the gut microbiome. Higher-intensity exercise has been associated with greater shifts in microbial diversity[10,12], which may amplify the production of short-chain fatty acids (SCFAs) and other metabolites that improve insulin sensitivity and glucose regulation[6,7]. In 2023, a study showed that exercise intensity elicited divergent changes in the gut microbiota composition among individuals living with T2DM[13]. Eight weeks of moderate-intensity continuous training and high-intensity interval training resulted in different alpha-diversity and relative abundance of specific health-promoting taxa between the intensity groups[13]. However, despite these microbial adaptations, no significant differences in glycemic outcomes or gut metabolites (including SCFAs) were observed between groups, and the mechanisms linking exercise intensity, gut microbial adaptations, and glycemic improvements remain poorly understood[13]. Modalities of exercise can have different but moderate impacts on overall human physiology and distinct microbial modifications of the gut, yet the evidence is limited when specifically considering gut microbial alterations to benefit T2DM[14]. While aerobic exercise is well-documented for its ability to enhance the abundance of SCFA-producing bacteria,

which are known to improve insulin sensitivity and reduce inflammation, resistance training may promote microbial diversity and complement aerobic exercise effects[10], suggesting that combined interventions could maximize the microbiota-mediated benefits for metabolic health[10,15,16].

To date, research has largely focused on isolated components of T2DM management, such as exercise-induced glycemic improvements or gut microbiome shifts, without fully integrating the findings into a cohesive framework. Investigating these relationships could provide valuable insights into optimizing exercise prescriptions and addressing inter-individual variability in T2DM management.

Objective: The primary objectives of this study are to determine whether performing combined aerobic and resistance training at a vigorous intensity significantly improves glycemia measured by hemoglobin A1c (HbA1c) and explore whether these changes are associated with an increase in gut microbial diversity compared to performing combined training at a moderate intensity. For the purpose of this analysis, a significant change in HbA1c will be considered a decrease equal to or exceeding the minimal clinically important difference (MCID) of 0.3%[17,18] following participation in the trial.

Hypothesis: We hypothesize that individuals exercising at a higher intensity will experience greater reductions in HbA1c and increases in gut microbial diversity than individuals training at a lower intensity.

Methodology

Study Design: The GUTFIT Study will be a parallel-group, single-blinded, randomized trial.

Study Setting: The GUTFIT Study will be conducted in the Cardiometabolic Exercise and Lifestyle Laboratory (CELLAB) in the Faculty of Kinesiology at the University of New Brunswick in Fredericton, Canada. This location was selected for its private gym facility, availability of necessary equipment, and access to research staff who are trained to work with individuals living with T2DM.

Sample Size: A power calculation was performed using G-power software (version 3.1.9.4, Germany) to determine the appropriate sample size for statistical significance. Based on an effect size of 0.30, an alpha of 0.05, and a power of 0.80, a sample size of n = 24 (n =12 per group) was found to be appropriate to detect a significant difference between intensity groups. To account for a potential dropout rate of up to 40%, a total of 40 participants (20 per group) will be recruited.

Inclusion Criteria: Participants will be eligible for inclusion if they are: 1) Community-dwelling adults aged 19 years and older, 2) currently living with T2DM, confirmed by a glycated hemoglobin (HbA1c) value of ≥ 6.5% or diagnosed by a physician with T2DM with an HbA1c of ≥ 5.7%, and 3) not currently partaking in regular physical activity, defined as 150 minutes of moderate-to-vigorous aerobic activity and two or more days of resistance training per week as per the 24-Hour Movement Guidelines[19], or averaging >10,000 steps per day over a five-to-seven-day period at baseline. To confirm and record participants physical activity levels, participants will complete the Get Active Questionnaire[20] and wear a Fitbit Charge 3 pedometer (Fitbit Inc., San

Fransisco, California, USA) for a 5–7-day period between the first and second baseline testing visits.

Exclusion Criteria: Participants will not be eligible to participate if they are: 1) diagnosed with low iron concentration, anemia, or currently being treated for these conditions, 2) diagnosed with any red blood cell-altering conditions, 3) diagnosed with any cardiovascular diseases that would impact the ability to safely participate in exercise training, 4) currently prescribed any medication that would impact the ability to use a heart rate monitor to accurately track exercise, 5) diagnosed with any gut microbiome-altering conditions (e.g., Celiac disease, GERD, irritable bowel syndrome (IBS), or inflammatory bowel disease (IBD)), 6) currently taking any gut microbiome-altering supplements (prebiotics/probiotics) or report taking antibiotics throughout the trial, or 7) have unstable T2DM medication over the past 3 months.

Recruitment: Participants will be recruited from the city of Fredericton and the Greater Fredericton Area using social media and radio advertisements, and advertisements placed in pharmacies, healthcare centers, physician offices, and community organizations. Further recruitment will occur through electronic communication, including e-newsletters within various organizations and groups. Participants from previous studies who expressed interest in being considered for future research will also be contacted.

Intervention: Eligible participants for the GUTFIT study will be randomized into one of two intervention groups: 1) moderate-intensity exercise, or 2) vigorous-intensity exercise.
Participants in both intervention groups will be scheduled to begin exercise sessions within 1 week of completing all baseline testing. The training protocol will last 16 weeks, requiring participants to complete two resistance-training sessions per week and at least two aerobic exercise sessions per week. All exercise sessions will be supervised by research staff and take place in an exercise facility located in the CELLAB. To maximize adherence to the intervention, exercise sessions are scheduled on a weekly basis with research staff available 7 days a week.

Resistance Training: The resistance training component of the intervention involves eight resistance exercises. The resistance exercises include seated chest press, triceps extension, lat pull down, leg press, knee extension, and knee flexion using Atlantis Strength weight machines, seated shoulder press using dumbbells, and unweighted abdominal crunches. Resistance training sessions will occur twice per week on non-consecutive days. The moderate-intensity group will perform resistance training at 65-70% of the one repetition maximum (1RM) for one set of 12-15 repetitions per exercise. The vigorous-intensity group will perform the resistance training at 75-80% of the 1RM for one set of 8-10 repetitions. Participants' prescribed weight for an exercise will be increased if they successfully complete the maximum number of repetitions of an exercise in two consecutive sessions. Abdominal crunches will remain unweighted throughout the duration of the intervention.

Aerobic Exercise: For the aerobic exercise component of the intervention, participants will expend 10 kilocalories (kcal) per kilogram of body weight per week (KKW) using a treadmill (StarTrac) or a stationary bike (Ergoline). Participants will be eased into the program using a 2-week progressive start; they will burn 8KKW in Week 1 and 9KKW in Week 2. For the remaining 14 weeks (Weeks 3-16), participants will expend 10KKW per week. Participants will be allowed to choose the number of sessions to complete their required aerobic exercise, with a minimum of two

days of exercise required and whether they complete a week of exercise using the treadmill or the bike. The speed and grade of the treadmill or the wattage and revolutions per minute (RPM) of the bike throughout the session will be determined by the participant as long as the prescribed intensity is achieved and maintained for the duration of each session. If necessary, the supervising research staff will instruct participants to adjust the speed or grade or watts or RPM to maintain the prescribed intensity. The moderate-intensity group will perform aerobic exercise at 45-55% of heart rate reserve (HRR), and the vigorous-intensity group will perform aerobic exercise at 70-80% of HRR. Participants will be given 5 minutes to warm up and reach target intensity at the beginning of each session, and 5 minutes for an active cool-down at the end of each session, neither of which will be counted toward their weekly caloric expenditure. To account for changes in cardiorespiratory fitness and ensure participants train at the appropriate intensity for their respective group, a new resting heart rate value will be measured at the start of every fifth week (Week 5, 9, 13) to account for changes in HRR.

<u>Caloric Expenditure:</u> Caloric expenditure will be calculated using the American College of Sports Medicine (ACSM) treadmill and stationary bike equations. Participants' weight will be recorded in kilograms at the beginning of each week and entered into training software to determine the total kcals that need to be expended for that week. Throughout each aerobic session, the participant's speed and grade for treadmill, or wattage and RPM for stationary bike, will be recorded every 5 minutes to calculate and track caloric expenditure and to determine the time required per session.

<u>Exercise Monitoring:</u> To ensure participants are exercising at the appropriate intensity, research staff will supervise each session and record participant heart rate every 5 minutes using the Polar Team2 (Polar, Kempele, Finland) system, along with treadmill speed and slope or bike wattage and RPM. The supervising staff will ensure that participants' heart rates remain within the prescribed intensity range, adjusting treadmill or bike settings as necessary.

<u>Resting Heart Rate Re-evaluation:</u> Resting heart rate (RHR) will be recorded at baseline testing and re-evaluated every 4 weeks throughout the intervention. As RHR will be used to prescribe intensity-dependent target heart rate ranges for the aerobic portion of the intervention, multiple measurements will be taken to find the average. Participants will be fitted with a Polar FT1 heart rate monitor (Polar, Kempele, Finland) worn around the chest and asked to lie supine on a massage table for a period of 10 minutes. Following 5 minutes of rest, heart rate, in beats per minute, will be recorded every minute for the next five minutes. These values will then be averaged and recorded as the RHR.

Primary Outcome*:* The primary outcome of the GUTFIT study is glycated hemoglobin (HbA1c) A finger prick will be conducted using a Safe-T Pro Plus single-use lancet (Accu-Chek, Roche Diagnostics, Switzerland) to collect a 1 microliter sample of whole blood. The sample will then be analyzed using a DCA Vantage Analyzer (V 4.4.0.0, Siemens Healthineers, Oakville, Ontario, Canada), a valid and accurate measure of HbA1c with intra-assay coefficients of variance of 1.55-2.29% [21], where rapid assessment will be conducted and result provided in approximately 6 minutes.

Secondary Outcomes: Anthropometric measurements will be taken over the span of two days, separated by less than one week. Participants height, weight, body mass index, and hip and waist circumference will be measured by a member of the research staff according to the Canadian Society for Exercise Physiology (CSEP) protocols[22]. Resting heart rate and blood pressure will also be measured following CSEP protocols using an Omron digital blood pressure monitor (OMRON Healthcare Co., Ltd., Kunotsubo, Terado-cho, Muko, Kyoto, 617-0002, Japan).

Body composition, including fat mass, lean mass, and body fat percentage, will be estimated using dual-energy x-ray absorptiometry (DXA) using a Hologic Horizon® DXA System (Hologic Canada ULC, Mississauga, ON, Canada). Participants will present to the CELLAB following a 12-hour overnight fast and will be asked to refrain from exercising for a 24-hour period prior to testing. Participants will also be instructed to wear loose-fitting clothing with no metal (buckles, zippers, etc.) and then lie supine on the DXA table and remain still for the duration of the scan. Arms will be placed at the participants' sides with palms facing medially and thumbs pointed upwards. For individuals too large for the width of the table, they will be positioned with their non dominant arm outside of the scan area and the results of the scanned arm will be duplicated.

Cardiorespiratory fitness will be assessed using a modified Balke and Ware treadmill test protocol with a progressive start. Participants will walk at a speed of 4.5 km/h at a 0% grade on a treadmill (9500HR (Life Fitness, Illinois, USA)) as a warmup. After 2 minutes, the speed will be increased to 5 km/h and the grade will increase to 2.5% (minutes 2-4). Following another 2 minutes, participants' speed will be increased to 5.5 km/h and grade to 5.0% (minutes 4-6). Grade will progressively increase by 1.0% every minute until a grade of 15% is achieved. If the participant has not fatigued, the speed will increase by 0.8 km/h each minute until test termination criteria are met. Following the completion of the test, participants will be instructed to continue walking at a decreased speed and grade for 5 minutes of active recovery. The participant will then have 3 minutes of seated passive recovery. Gas exchange will be continuously monitored using a TrueOne 2400 Metabolic Cart (ParvoMedics, Salt Lake City, Utah, USA), heart rate data will be obtained over the course of the test using the Polar FT1 heart rate monitor (Polar, Kempele, Finland), and blood pressure data will be recorded every 2 minutes using a SunTech® Tango M2 Automated Blood Pressure Monitor (SunTech Medical, Inc., Morrisville, North Carolina, USA).

Muscular strength will be assessed through 1-repetition maximum (1-RM) testing. Participants will perform two warmup sets – the first with a weight they can complete 6-10 repetitions of the exercise with, and the second using a weight they can complete 3-5 repetitions with. Following the warmup sets, research staff will increase the weight until participants can no longer lift the weight or show improper form in the movement. Rest periods between maximal attempts will be 1-2 minutes.

The gut microbiome samples will be collected using OMNIgene®•GUT fecal sample collection kits (DNA Genotek Inc., Ontario, Canada). Participants will be provided detailed instructions on how to collect a sample and then provided with a collection kit to use at home before their first exercise sessions and after their last exercise session. Once collected, samples will be pipetted into microcentrifuge tubes for storage at -80 degrees Celsius until sent to a partner site for analysis using next-generation sequencing (Illumina MiSeq) of 16S ribosomal DNA genes.

Additionally, at baseline and post-testing, *quality of life* will be assessed using the Audit of Diabetes-Dependent Quality of Life (ADDQOL) and SF-36, *dietary habits* will be recorded for a weekday and weekend day using the ASA 24, and *eating behaviour* will be assessed through the Three-Factor Eating Questionnaire-R-18 (TFER-R18). *Sleep quality* will be measured through the Pittsburgh Sleep Quality Index (PSQI) and *traits of masculinity and femininity* will be recorded using BEM's Androgyny test and the Sex and Gender-Based Analysis (SGBA).

Blinding: To maintain single blinding, participants will remain unaware of their randomizations to the moderate- or vigorous-intensity exercise group for the duration of the intervention.

Randomization: Randomization of the intervention participants will occur following completion of the second baseline testing. visit using a 1:1 allocation ratio for every four males or females in a balanced randomized block design performed in sealed envelope software[23]. A member of the CELLAB staff who is not related to the project and has no contact with participants will hold the password-protected randomizations. When a new participant completes baseline testing and is ready for randomization, the participants ID number and sex will be sent to the member of staff with randomizations via email and they will respond to research staff with the randomization (moderate or vigorous) for that participant.

Data Collection and Management: At the time of first contact with research staff, participants will be assigned a unique identifier (ID), and files will be deidentified. Participants will meet with research staff for the purpose of data collection a total of four times: twice at baseline testing and twice at post-testing. All data obtained from baseline and post-intervention testing visits will be collected in written form and then transferred to electronic files. All other data collected throughout the intervention will be collected electronically. Physical versions of files will be stored locally in a locked cabinet in a locked room in a restricted research lab (CELLAB), while digital files will be password-protected on a computer that will only be accessible by the primary investigator and research staff.

Informed Consent: Prior to the first baseline testing visit, eligible participants will be provided with a digital copy of the consent form to review. The consent form described in detail the procedures of this study and any benefits and risks associated with their participation. At the beginning of the first testing visit, participants will have time to review a physical copy of the consent form, ask any questions, and consider their participation. If the participant decides to proceed with participating in the study, they will be asked to provide written consent by signing the form, which will be cosigned by research staff. All participants are free to withdraw from the study at any time.

Dissemination: Results from the GUTFIT study will be submitted to peer-reviewed journals and presented at scientific meetings. The findings from this study will be used to support and drive future randomized trials exploring the impact of exercise intensity on the gut microbiome for individuals living with T2DM. Once data analysis has been completed, feedback of the research findings will be provided to the participants, in plain language, if desired. Furthermore, participants may request copies of the journal publications or conference abstracts/proceedings that result from the research they have participated in.
